# Suppression of thermo-acoustic instabilities in horizontal Rijke tube using pulsating radial jets

**DOI:** 10.1016/j.mex.2023.102325

**Published:** 2023-08-15

**Authors:** Nilaj N. Deshmukh, Afzal Ansari, Asim P. Tajir, Craig C. Almeida, Ankita S. Shetty, N.S. Danie, Suchita K. Kadam

**Affiliations:** a Department of Mechanical Engineering, Fr. C. Rodrigues Institute of Technology, Sector- 9A, Vashi, Navi Mumbai 400703, India; b Ramrao Adik Institute of Technology, Nerul, Navi Mumbai 400 706, India

**Keywords:** Thermo-acoustic instabilities, Rijke tube, Active control, Pulsatile injection, Closed-Loop Active Control Method for Suppression of Thermo-Acoustic Instabilityusing Pulsating Radial Jets

## Abstract

Thermo-acoustic instability has been observed in gas turbines, rocket engines, and aero-engines. Acoustic perturbations grow and change the characteristics of the flow due to instability. The present work describes the use of pulsating air jets to suppress the thermo-acoustic instabilities. In present study pulsatile micro-jets are placed downstream of the burner radially which breaks the coupling between acoustic waves and unsteady heat release.

A microphone connected to LIFA (LabVIEW Interface for Arduino) was used to detect the sound pressure levels. By controlling the airflow rate of the pulsatile jets, the sound pressure levels were suppressed down to the background noise level using minimum energy and time. A closed-loop control system is developed for this purpose, which works on the feedback signal acquired from microphone. To simulate the one dimensional combustion phenomenon, an experimental setup called Rijke tube was used. The suppression was most effective for the pulsatile jets of 27-33 Hz pulsation frequency range and at a flow rate of 6.8 LPM. This control strategy effectively controlled the combustion instability of around 35-42 dB.

• The closed loop control method is built on DAQ and Arduino using the LabVIEW interface for Arduino (LIFA).
• Developed closed loop active control method was observed to be effective for suppression of thermo-acoustic instability.
• Optimum position of the radial planes of micro-jets with respect to the burner was decided to improve the efficacy of the pulsatile jets towards suppression of thermo-acoustic instability.

## Graphical abstract

**Figure:**
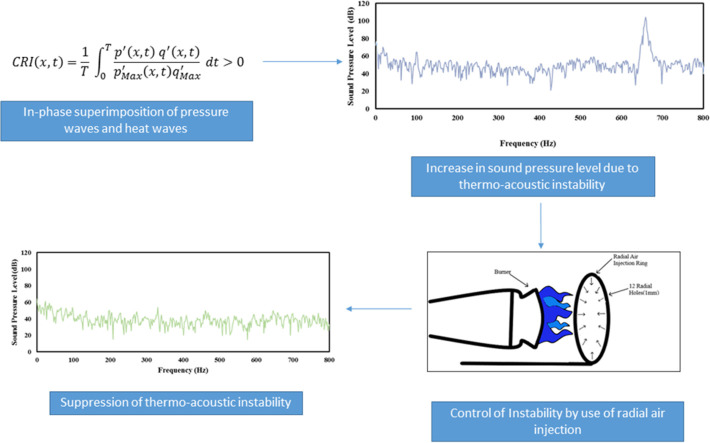

Specifications table

**Table:** 

Subject area:	Thermo-acoustics
More specific subject area:	Control of Thermo-acoustic Instability
Name of your method:	Closed-Loop Active Control Method for Suppression of Thermo-Acoustic Instabilityusing Pulsating Radial Jets
Name and reference of original method:	*N. N. Deshmukh, A. Ansari, P. Kumar, A. V. George, F. J. Thomas, and M. S. George, Effect of position of radial air injection plane on control of thermo-acoustic instability using active closed-loop method, JVC/Journal Vib. Control, August, 2021* https://doi.org/10.1177/10775463211050175
Resource availability:	*The LabVIEW .lvm files for data acquisition of sound pressure level and excel files used for post processing can be made available on request.*

## Method details

Occurrence of thermo-acoustic instability in gas turbine and rocket engines have been serious issue due to which defence and space programs as well as the energy and aviation industries are more concerned towards resolving the issues [1,2]. The developed high amplitude pressure fluctuations due to thermo-acoustic instabilities are responsible for enormous vibrations, causing structural damage to mechanical parts and failure of electronic systems in the vehicle and the satellite [3,4]. The dynamic behaviour of the combustion instability in a lean premixed gas-turbine combustor was investigated experimentally using nonlinear dynamics, with a focus on how the dynamic properties of the pressure fluctuations change as the equivalence ratio increases [5]. Forecasting the conditions responsible for combustor's dynamic behaviour in the early stages of design is major challenge [6]. Extensive research has been carried out to detect and control these instabilities. However, achieving this phenomenon experimentally is very difficult. As a result, fundamental research on thermo-acoustic instability began on Rijke tube, which is prototypical system to study thermo-acoustic instability [7], [8], [9], [10]. The Rayleigh criterion is a standard tool for predicting and investigating combustion instabilities in experimental and numerical studies [11], [12], [13], [14]. The Rayleigh Index is a diagnostic tool for detecting thermo-acoustic instability. The performance of the thermo-acoustic mechanism is influenced by many parameters, particularly the heat release rate, perturbation at the flame [15] and the time delay between acoustic perturbations at the fuel injector [16].

Due to adverse effect of thermo-acoustic instabilities on the performance of power producing devices, there is a need for developing early warning device and implement appropriate control strategies [17]. The control strategies are categorised in two main approaches namely active control and passive control. Changes to the structure and geometry of the combustor are examples of passive control techniques, however, these methods are effective for a limited frequency range [18]. It was possible to decrease and narrow the frequency band at one operating condition by adjusting various controlling factors [19]. The disadvantage of these control methods is that the changes are typically rigid and difficult or expensive to implement. This makes passive control very inflexible, allowing only a narrow range of applications [20]; some passive control techniques include the use of Helmholtz resonators [21,22], perforated plates, acoustic liners [22], half-wave and quarter-wave tubes [23,24].

Active control techniques are more popular due to their wide range of operational flexibility [25,26]. The primary goal of active control strategies is to disrupt the interaction of uneven heat release and acoustics within the Rijke tube [3]. Few active control methods for effective control of thermo-acoustic instability include radial air injection [27,28], fuel flow modulation [29], fuel injection [30,31,30] and use of loudspeakers [18,32]. Active control techniques, automatically adjusts itself to the presence of instabilities and can trigger as well as modulate the controller by itself, hence eliminating chances of human intervention and error, which can often lead to the blowing off of the flame. A systematic control design based on the interactions between the combustor and the control system over a wide frequency range that takes into account all of the combustor's dominant dynamic behaviour, including thermo-acoustic resonance, is thus required and was adopted, which results in better performance [30]. The actuation of this external source of energy can be either in a closed-loop or open-loop system [13]. The main advantage of active control techniques over passive ones is the flexibility and adaptability of the control system to dynamic environments and excitations. Active control techniques have also shown higher run-time efficiencies.

An open loop control method was developed to suppress thermo-acoustic instabilities [33]. This method involves a steady radial air injection near the flame and able to suppress instabilities up to background noise. The airflow to the ring was regulated through a rotameter and supply of air is continuous and maintained by operator based on instability. So, this type of open-loop technique requires human interference and motivation to develop closed-loop strategies in terms of responses obtained. In an open-loop technique, the air supplied to the injector plane is constant, resulting in energy waste, whereas, in a closed-loop method, only a sufficient amount of air is supplied based on sensor feedback. When additional air is supplied to the combustion chamber in an open-loop technique, the system's weight increases, and the flame is sometimes extinguished. To overcome this problem of an open-loop system, a closed-loop active control system with radial air injection to suppress thermo-acoustic instabilities has been developed by Deshmukh et al. [28,34]. The feedback from the microphone was used to control the supply of air. As a result, the sophisticated system can be utilized successfully in any operation without human interference. The present research aims to remove the requirement for an additional supply of air which was supplied to the radial micro-jet from the compressor. The pulsatile flow in the form of radial micro-jet introduces with the help of a reciprocating mechanism near the flame zone. The reciprocating mechanism takes air from the burner's face and returns it to the same location at the set frequency. As a result, the pulsating mechanism requires no additional air to generate air micro-jets. Therefore, the proposed technique reduced thermo-acoustic instabilities without adding any extra air to the combustor.

To carry out experiment at laboratory, a tube of 800 mm length and 80 mm diameter was used as 1D combustor. Based on previous experimental results, the burner position was set at a location of x/L = 0.25, where x is the position of the burner in the Rijke tube and L is the total length of the Rijke tube which is shown in Fig. 2(a). The burner is used with radial air injection ring, which is positioned horizontally and coaxially within the Rijke tube as shown in Fig. 2(b). The 30 mm diameter burner has 60 holes. The experiments were carried out for various distances between the burner head and the radial air injection plane. The compressed air is dried and placed in the reserve tank. The gas used for combustion is LPG, its characteristics are given in Table 1.

**Table 1:** Characteristics of LPG.

Sr. No.	Property	Data
1	Chemical Formula	Mix of mainly C_4_H_10_& C_3_H_8_
2	Boiling Point	20°C to -27°C
3	Explosive Limit	1.5-9.0
4	Vapour Density(Air = 1)	1.8
5	Specific Gravity(Liquid)	0.53-0.54
6	Appearance	Colourless
7	Odour	Odourless
8	Physical State	Compressed Liquid
9	Vapour Pressure at 40°C	1050 KPa (Max)

The stoichiometric fuel to air ratio for LPG combustion in air is between 15.6:1 and 15.8:1. But lean premixed combustion can be possible up to a fuel to air ratio of 50:1 to 60:1.

**Fig. 1:**
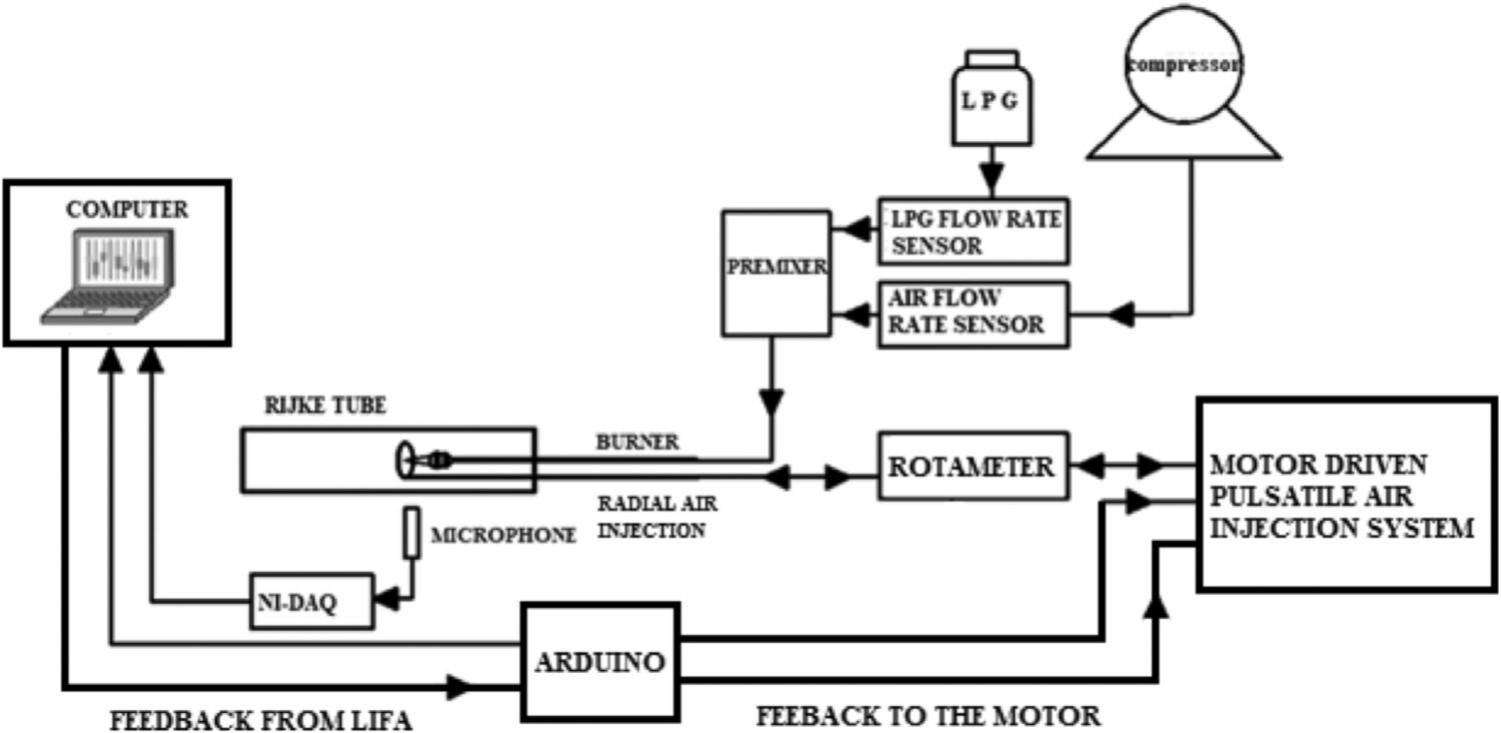
Schematic diagram of the experimental setup.

The flow rates of air and LPG was controlled and monitor using rotameters. Air and LPG are mixed as per requirement in premixer. The premixer ensures that LPG and air are properly mixed before entering to the burner. The schematic of experimental set-up is shown in Fig. 1, and the photographic view of the experimental setup is shown in Fig. 3.

**Fig. 2:**
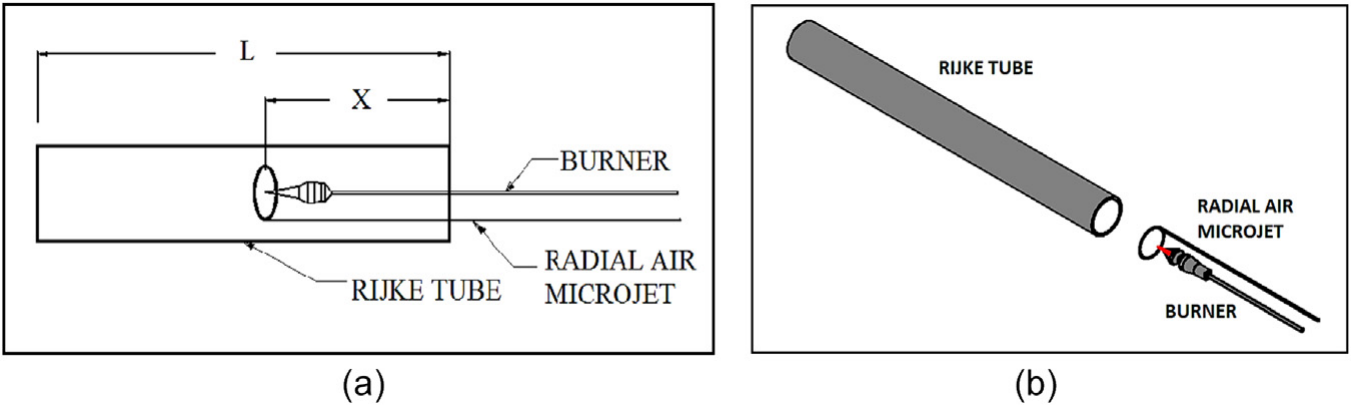
(a) The letter ‘X’ denotes the location of the burner in the Rijke tube, and the letter L denotes the overall length of the Rijke tube. (b) Shows the arrangement of a radial air micro-jet and a burner with a Rijke tube.

**Fig. 3:**
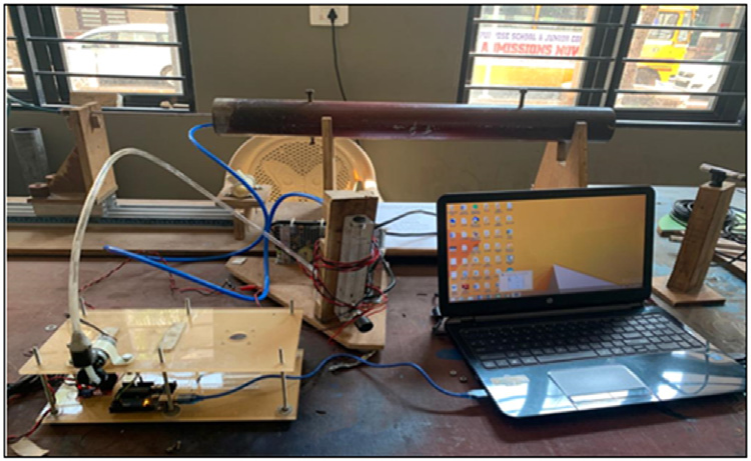
Photographic view of the experimental setup.

### Control technique

Pulsating air in the form of jets is introduced near the flame with the help of radial injection ring. The inner diameter of the copper tube is 5 mm. A bending machine was used to bend the copper tube into a ring shape. Twelve circumferentially equidistant holes of 1 mm each are drilled into the copper ring.

**Fig. 4:**
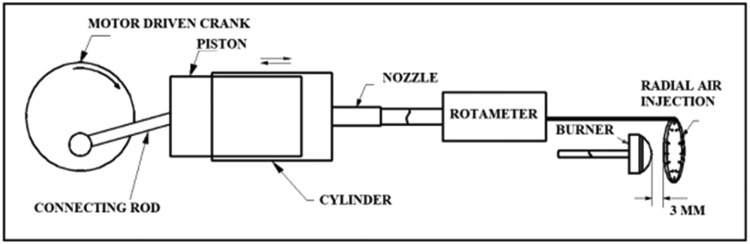
Reciprocating Mechanism for Pulsatile air.

The copper ring arrangement was clamped to the burner air-fuel supply pipe. This clamping method ensured that the copper ring could be moved axially inside the Rijke tube, hence providing flexibility in its placement relative to the burner head. For this study, the planar distance between the burner head and the copper ring was set at 3 mm, which is shown in Fig. 4.

### Pulsatile setup

By using a reciprocating mechanism, pulsatile air was used to eliminate instabilities. These air pulsations were created using a piston-cylinder arrangement. The piston, cylinder, connecting rod, and crankshaft are illustrated in Fig. 4.

The selection of the pulsatile device was made in such a way that it should not produce pulsations with a high velocity, which would result in the extinguishing of the flame while keeping the frequency in a suitable range. A variable-speed electric motor is used to give power to the crankshaft. The Arduino is used to control the speed of motor based on the thermo-acoustic instabilities. The pulsating air jets were created by a small piston-cylinder arrangement with a piston diameter of 14 mm and a bore diameter of 16 mm. The crankshaft utilized has a stroke length of 14 mm. The motor can run up to 10,000 rpm, and the geared assembly helps give a maximum crank speed of 3000 rpm, or about 50 pulses of air per second. A 12V DC motor powers this arrangement with a gear reducer. This motor was connected to an Arduino microcontroller and an LN298 motor driver. It is linked to LabVIEW Software via the LabVIEW Interface for Arduino (LIFA). The reciprocating action of the piston created these pulsating air jets. The nozzle at the top of the cylinder was connected to the open end of the copper tube via a ½-inch PVC pipe. The piston has inlet valves which open during the suction stroke and air can be sucked easily from the atmosphere. The inlet valve closes during the compression stroke which results in the air to pass through the radial holes in the copper ring resulting in pulsatile jets. The flowrate of the air through the copper tube creating the pulsatile jet was calculated using the following relations.

Mean piston speed is given by

(1) $$V_{mean}=\frac{2ln}{60}\left(m/s\right)$$

The air flow rate is calculated using the following formula:

(2) $$\dot{m}=\rho \frac{\pi }{4}D^{2}L\frac{N}{60}\;\left(\text{kg}/s\right)$$

Where,

ρ= density of air
D = bore of the reciprocating cylinder
L = stroke length of the cylinder
N = revolutions per minute.

The flow rate is controlled using a motor driver coupled to a microcontroller which decides whether the rpm has to be increased or decreased based on the feedback received from the LIFA unit.

### Block diagram of the algorithm

The system starts with controlling Arduino with the help of LabVIEW. INIT is a block function that sets-up a connection to an Arduino running the LabVIEW Interface for Arduino Sketch. After initializing the Arduino, its pins are initialized. Two digital pins are initialized as OUTPUT pins, and one digital pin is initialized as PWM (Pulse Width Modulation) OUTPUT signal. These pins are used to control the speed of the motor using the L298N 2A motor driver. The motor is powered using a 12V SMPS. The motor is started at a 30% duty cycle initially. DAQ Assistant is used to get sound pressure in Pascal. The observed sound pressure value is compared with the set cut-off value to check for instability in the system. If the observed value is greater than the cut-off value, then the instability is present.

**Fig. 5:**
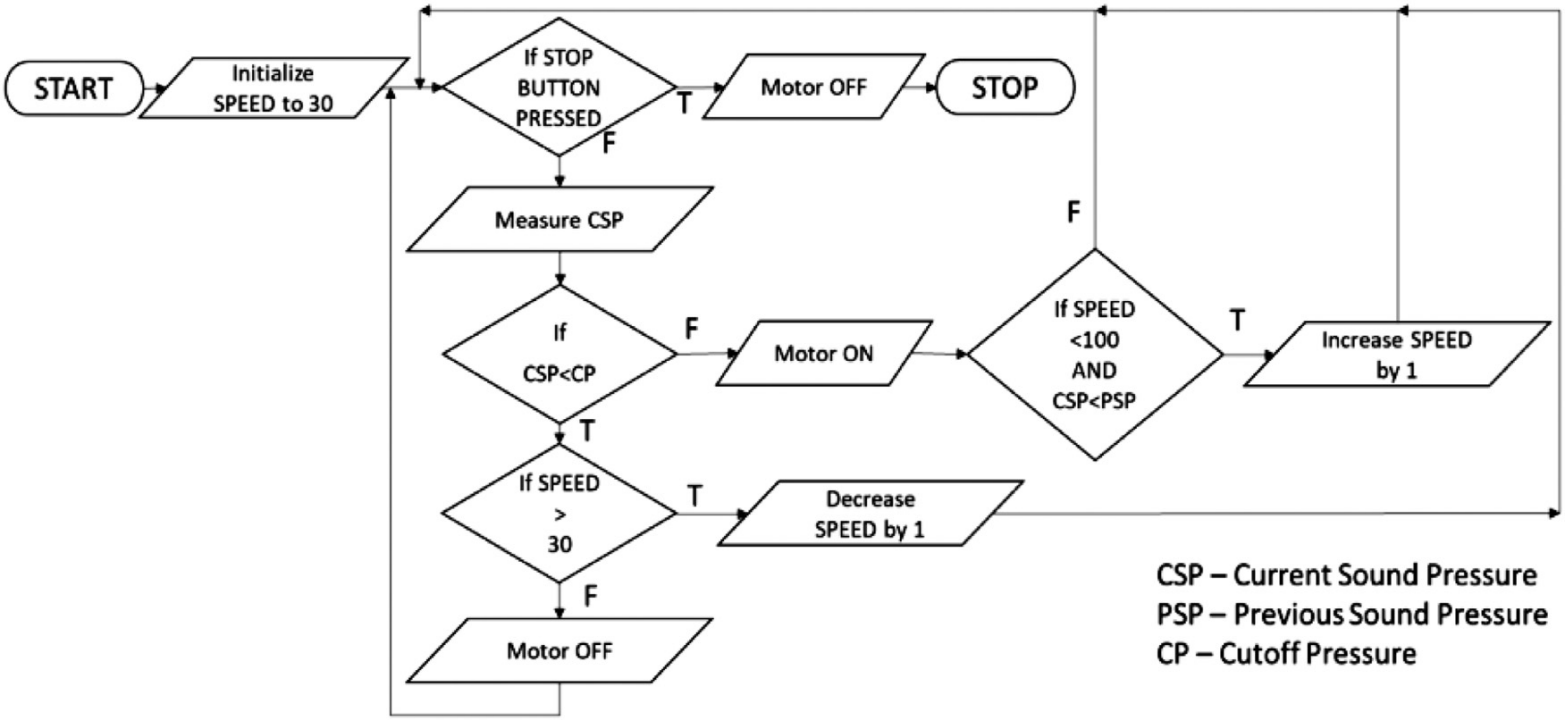
Flow chart of the algorithm used to generate pulsating jets.

Based on the feedback from the microphone, if the instability is present, the motor is switched ON at the initial duty cycle, i.e., 30%. Then the current sound pressure value is compared with the previous value, and if the current value is less, then it is assumed that the suppression is working, and hence the speed is increased by one duty cycle for each loop iteration, as shown in Fig. 5.

The same speed is maintained for the next loop iteration. If instability is not present or suppressed, then the motor's current speed is checked. If it is higher than the initial duty cycle value, i.e., 30%, then the speed is decreased by one after each loop iteration. Once the speed becomes equal to the initial duty cycle, i.e., 30%, the motor is switched OFF. This process keeps on repeating until the STOP button is pressed.

### Instrumentation

The mass flow of air and LPG to the burner was measured and manually controlled using a 20 LPM rotameter and 1 LPM rotameter, respectively. The flowmeter used is Omron MEMS flow meter (model D6F-20A6-000) with a measurement range of 0 to 20 LPM. The calibration for the same is given on supplier website. An Onosokki condenser-type microphone (MI-1433) with a frequency range of up to 8 kHz is used to measure the instabilities. The microphone used is Onosokki MI-1433 which can measure noise with frequency range of 20 Hz to 8 kHz. Its sensitivity is -29 dB ±3 dB re. 1 V/Pa 36 mV/Pa (1 kHz) with capacitance of 13 pF. It has an operating temperature range of −10 to +50 ℃… It is coupled with the MI-3111 microphone preamplifier with a convenient BNC-type connector. The microphone is interfaced with LabView software using the NI-9234 module mounted on the NI-DAQ-9172 chassis. The output from the microphone is conditioned and sent to LabView via this module. As the microphone was placed outside of the Rijke tube, it will be unaffected by the node and antinode pressure locations.

### Experimental methodology

The zones of thermo-acoustic instabilities exist only at specific positions of the heat source inside the Rijke tube. The experiments were carried out with an air-fuel ratio of about 30:1 and an equivalence ratio of 0.93. In preliminary experiments, no instability was observed up to an equivalence ratio of 1, and at an equivalence ratio of 0.65, the flame was blown off. Strong instability was observed at an equivalence ratio of 0.93, so this condition was chosen for the experiment, with the flow rate set at about 15 LPM and the LPG gas flow set at about 0.5 LPM. The distance measured from the burner head to the plane of the copper ring was fixed at 3 mm and was kept constant throughout the experiment. The position of the burner was to start from x/L= 0 to x/L = 0.25, where x is the location of the burner in the Rijke tube, while L is the overall length of the Rijke tube. A method used to find these positions was to start from the location of the burner, measured from the left side of the Rijke tube to the overall length of the Rijke tube. The fundamental frequency was approximately 225 Hz, as calculated by the formula C/2L (in which C is the speed of sound and L is the length of the Rijke tube used, which was 0.8 m). The details of calculations are as follows:

The acoustic velocity (C) can change according to the temperature of air inside the Rijke tube. The length (L) is in fact the effective length of the Rijke tube which is given by the following formula:

$$L_{\text{eff}}=L_{\text{actual}}+0.6D_{\text{inner}}$$

where,

L_eff_ = Effective length of the Rijke tube (used in the formula for calculating fundamental frequency)
L_actual_ = Actual length of the Rijke tube
D_inner_ = Inner diameter of the Rijke tube
Sample calculation
For the experimental setup used in the lab
L_actual_ = 800 mm
D_inner_ = 80 mm
At 363 K (90°C) acoustic velocity (c) = 382 m/s
L_eff_ = L_actual_ + 0.6 D_inner_ = 848 mm = 0.848 m
Fundamental frequency (f) = C/L_eff_ = 225.28 Hz

Experimental results shows the observed instabilities of 3rd mode (3×225 = 675 Hz). Thus, the frequency of the observed thermo-acoustic instability lies within the 670–690 Hz range. The sound pressure readings were obtained via the microphone in Pascal's and plotted. They were conditioned using LabVIEW Software and converted from a time domain signal to a frequency domain signal, providing more information about the signal. The positive peak readings were obtained by the microphone via the DAQ and were sent to the LabVIEW. The values were obtained for the 3^rd^ mode displayed on the LabVIEW interface in decibels (dB). The software then triggers the control system, starting from a minimum speed and gradually increasing until the instabilities are suppressed. The speeds are restricted to prevent flame blow-off. The expected time required to control the thermo-acoustic instabilities should be minimum and flow requirement should be also minimum, which reduces the extra addition of weight in the combustor.

### Measurements

The sound pressure level was measured using the previously mentioned Onosokki condenser microphone. The microphone was placed about 150 mm from the end of the Rijke tube and at a 30^o^ offset from the axis of the tube. This placement ensured the closest possible placement of the microphone without exposing it to the risk of damage due to a larger, fuel-rich flame. Sampling time for acquiring sound pressure level was 20 second and sampling rate was 100 ks/s during the all experiments.

### Method validation

**Fig. 6:**
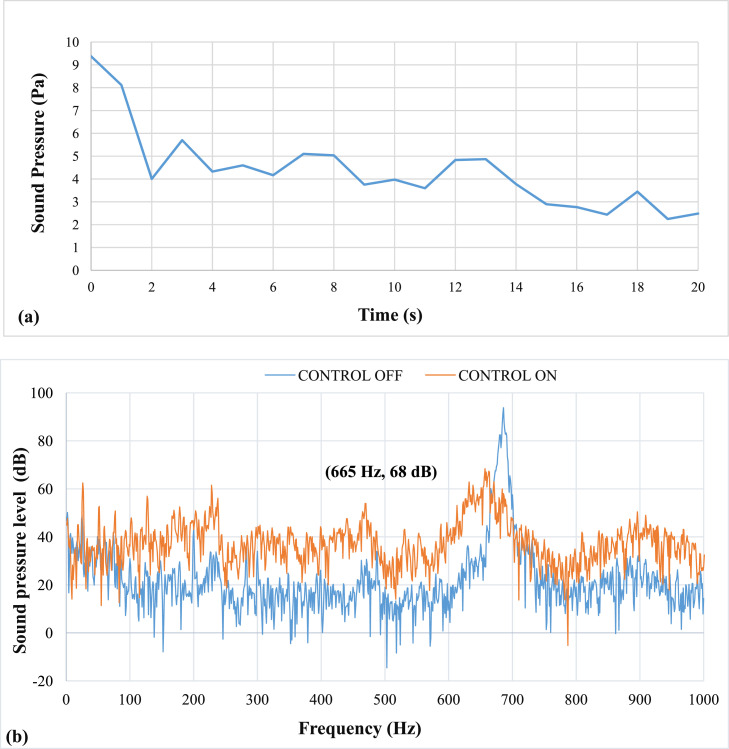
Results of Pulsation frequency of 20 Hz (a) Positive Peak Obtained and (b) Frequency Spectra.

The results of the experiment on the effects of pulsating air jets on the thermo-acoustic instabilities are as follows: All results were recorded while the copper ring was fixed at 3 mm from the burner head. Fig. 6 shows results of pulsation frequency of 20 Hz. The bold black parentheses in the sound pressure level spectrum in Fig. 8, Fig. 9, Fig. 10, Fig. 11, Fig. 12, Fig. 13 represent the peak value of the control on process. It shows the value after the suppression which is visible on the graph as reduced amplitude of the peak value compared to the case when control is off.

Fig. 6 shows results of pulsation frequency of 20 Hz. The results at an applied pulsation frequency of 20 Hz show that the air pulsations were insufficient in reducing the thermo-acoustic instabilities. The lag, or delay period between the instability activation and damping, is 2 seconds. It also takes more than 20 seconds to lower the sound pressure. However, it was noticed that there was a drop of about 25 dB in sound pressure level (SPL). The airflow rate at this speed was calculated to be approximately 5 LPM. The mean piston velocity was found to be 0.66 m/s.

**Fig. 7:**
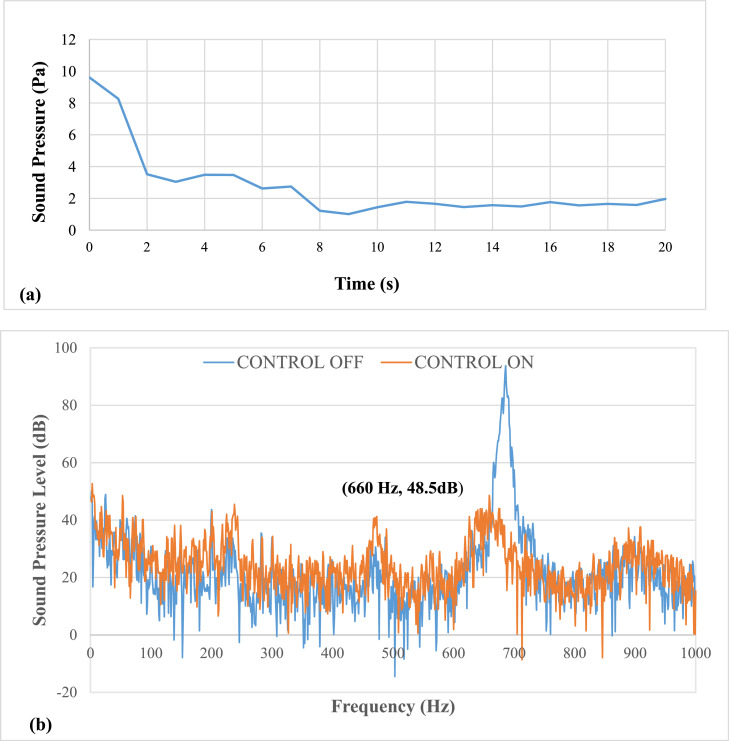
Results of Pulsation frequency of 27 Hz a) Positive Peak Obtained and b) Frequency Spectra.

Fig. 7 shows results of pulsation frequency of 27 Hz. The results show that at an applied pulsation frequency of 27 Hz, the air pulsations efficiently eliminate thermo-acoustic instabilities. The lag, or time between the activation and damping of the instability, is 3 seconds. The entire time required to eliminate instability is ten seconds. It was noticed that there was a drop of about 42 dB in sound pressure level (SPL). The airflow rate at this speed was calculated to be approximately 6.1 LPM. The mean piston velocity was found to be 0.88 m/s.

**Fig. 8:**
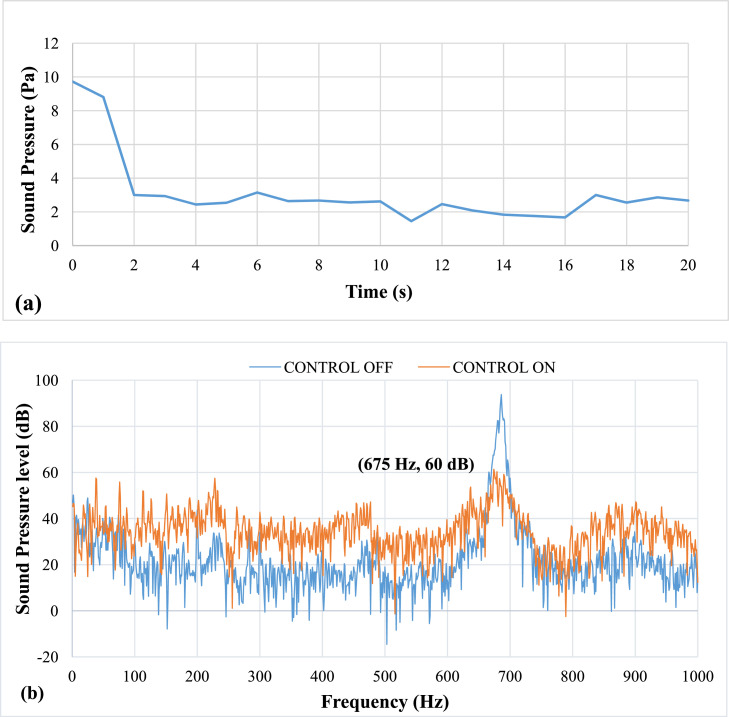
Results of Pulsation frequency of 33 Hz a) Positive Peak Obtained and b) Frequency Spectra.

Fig. 8 shows results of pulsation frequency of 33 Hz. The results show that at an applied pulsation frequency of 33 Hz, the air pulsations effectively reduce the thermo-acoustic instabilities. The lag, or time between the activation and damping of the instability, is 2 seconds. The entire time required to remove the instability is 11 seconds. However, it was noticed that there was a drop of about 35 dB in sound pressure level (SPL). The airflow rate at this speed was calculated to be approximately 6.8 LPM. The mean piston velocity was found to be 1.01 m/s.

**Fig. 9:**
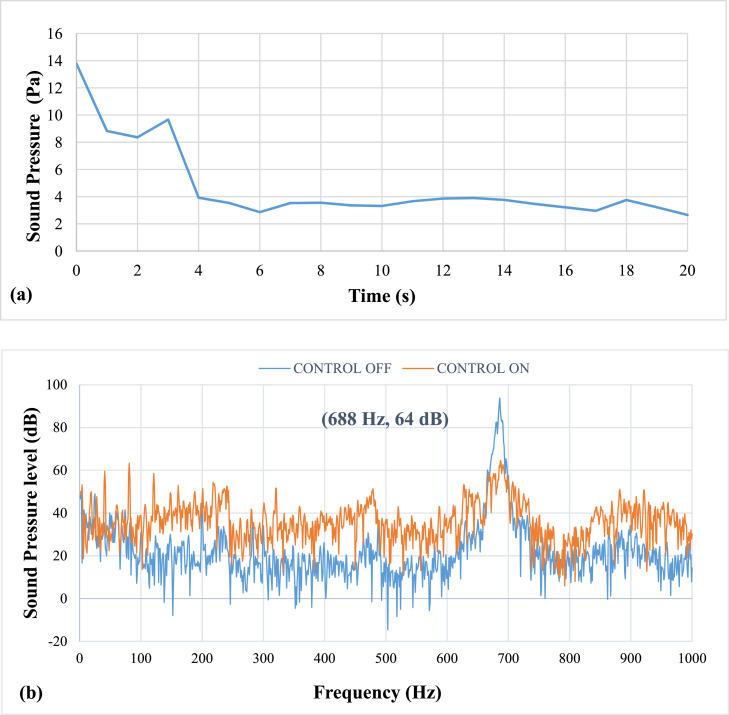
Results of Pulsation frequency of 39 Hz a) Positive Peak Obtained and b) Frequency Spectra.

Fig. 9 shows results of pulsation frequency of 39 Hz. The results show that at an applied pulsation frequency of 39 Hz, the air pulsations were ineffective in suppressing the thermo-acoustic instabilities. The lag, or time between the activation and damping of the instability, is 4 seconds. The entire time required to remove the instability is 6 seconds. It could not eliminate the instabilities and noticed a drop of about 32 dB in sound pressure level (SPL). The airflow rate at this speed was approximately 7.6 LPM. The mean piston velocity was found to be 1.14 m/s.

**Fig. 10:**
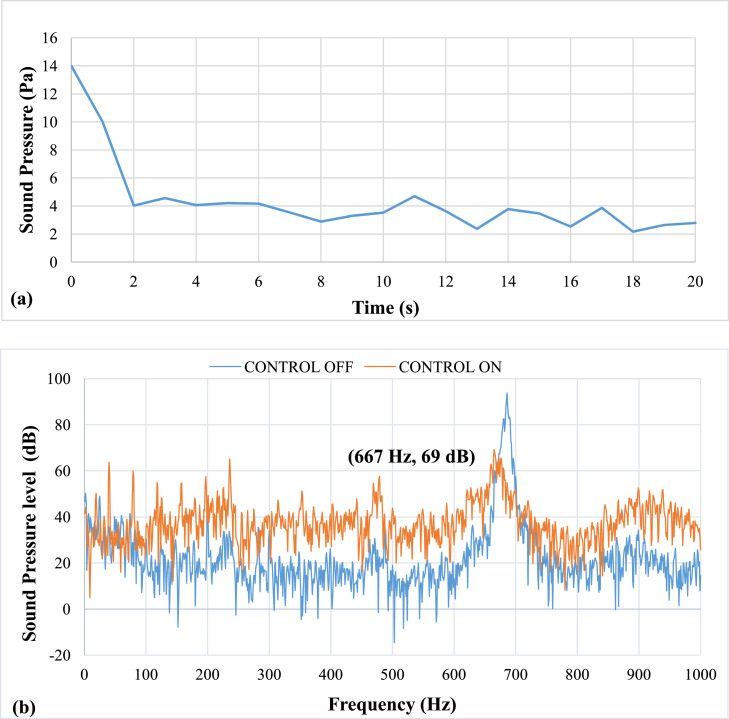
Results of Pulsation frequency of 44 Hz a) Positive Peak Obtained and b) Frequency Spectra.

Fig. 10 shows results of pulsation frequency of 44 Hz. The results show that at an applied pulsation frequency of 44 Hz, the air pulsations were ineffective in suppressing the thermo-acoustic instabilities. The lag, or time between the activation and damping of the instability, is 2 seconds. The entire time required to remove the instability is 14 seconds. It could not fully eliminate the instabilities and noticed a drop of about 29 dB in sound pressure level (SPL). The airflow rate at this speed was calculated to be approximately 8.3 LPM. The mean piston velocity was found to be 1.25 m/s.

**Fig. 11:**
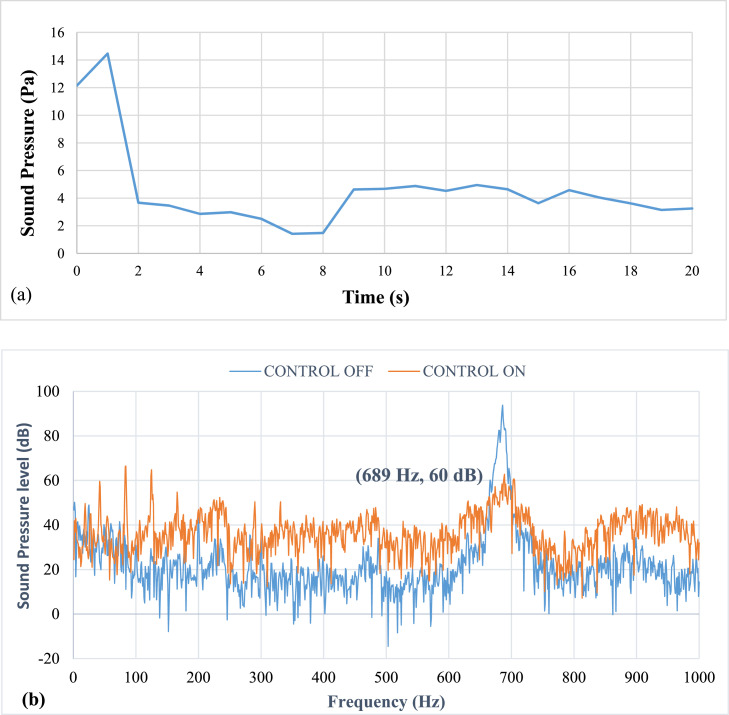
Results of Pulsation frequency of 49 Hz a) Positive Peak Obtained and b) Frequency Spectra.

Fig. 11 shows results of pulsation frequency of 49 Hz. The results at an applied pulsation frequency of 49 Hz show that the air pulsations were ineffective in suppressing the thermo-acoustic instabilities. The lag, or time between the activation and damping of the instability, is 2 seconds. The entire time required to remove the instability is 8 seconds. It could not fully eliminate the instabilities and noticed a drop of about 28 dB in sound pressure level (SPL). The airflow rate at this speed was calculated to be approximately 9.3 LPM. The mean piston velocity was found to be 1.41 m/s.

**Fig. 12:**
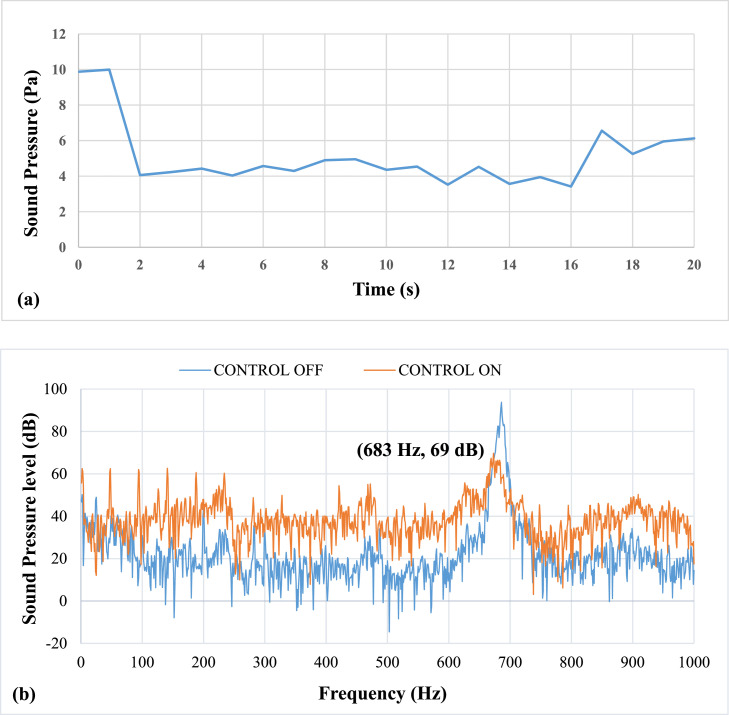
Results of Pulsation frequency of 53 Hz a) Positive Peak Obtained and b) Frequency Spectra.

Fig. 12 shows results of pulsation frequency of 53 Hz. The results at an applied pulsation frequency of 53 Hz show that the air pulsations were ineffective in suppressing the thermo-acoustic instabilities. The time between the initiation and damping of the instability is 2 seconds. The total time needed to eliminate the instabilities is 16 seconds. It could not fully eliminate the instabilities and noticed a drop of about 24 dB in sound pressure level (SPL). The airflow rate at this speed was calculated to be approximately 9.75 LPM. The mean piston velocity was found to be 1.54 m/s. The flame also happened to blow off on two occasions.

**Fig. 13:**
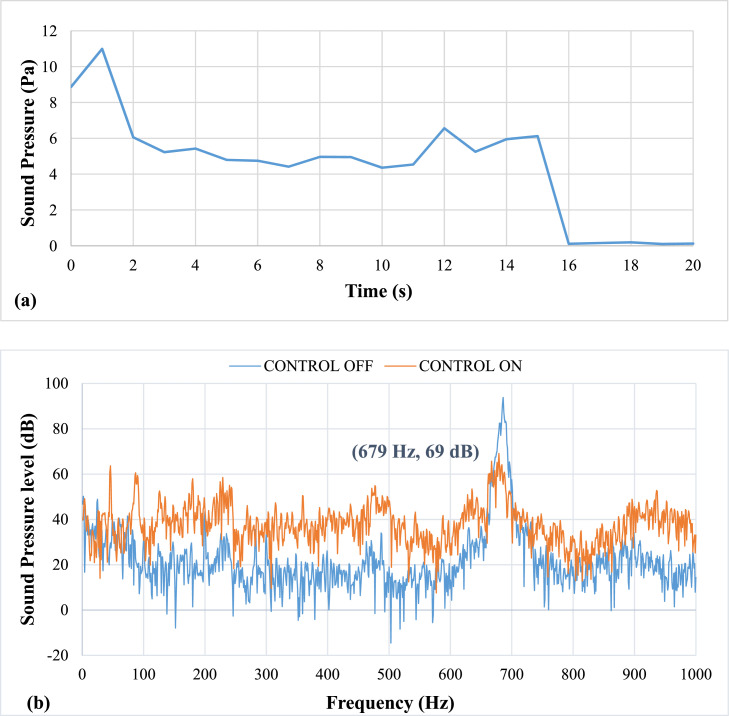
Results of Pulsation frequency of 59 Hz a) Positive Peak Obtained and b) Frequency Spectra.

Fig. 13 shows results of pulsation frequency of 59 Hz. The results at an applied pulsation frequency of 59 Hz show that the air pulsations were powerful in suppressing the thermo-acoustic instabilities. The time between the initiation and damping of the instability is 2 seconds. The total time needed to eliminate the instabilities is 16 seconds. However, it was noticed that there was a drop of only about 23 dB in sound pressure level (SPL). The airflow rate at this speed was calculated to be approximately 10.5 LPM. The mean piston velocity was found to be 1.71 m/s. It was also noticed that the flame was blown off at this speed setting.

### Analysis of the results

**Fig. 14:**
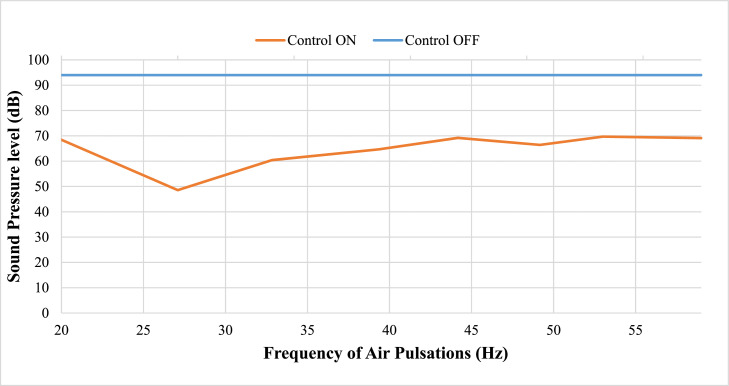
Comparative Plot at varying speeds of Air Pulsations.

The earlier section in Fig. 6 to Fig. 13 shows a graphical representation of the various frequency spectra, demonstrating that as pulsating air jets interact with the flame, unsteadiness in acoustic pressure is dampened. Fig. 14 shows a comparison plot of the effect of pulsating air jets at various speeds on thermo-acoustic instabilities. The time required to suppress the acoustic pressure depends on the frequency of pulsating jets. It has been tried to evaluate and develop a control technique for eight various frequencies of pulsating micro-jets in this study. The time required to control thermo-acoustic instabilities varies with frequency. Thus, the frequency of pulsating jets is critical for controlling thermo-acoustic instabilities in a short time. The suppression was observed to be most effective between pulsation frequencies of 26 and 33 Hz and at a flow rate of 6 LPM. Air pulsations below this speed were simply insufficient to eliminate instabilities, while those beyond this speed could only partially remove instabilities. Because of the low frequency, the flame moves; as a result, harmonic mode appears regularly, causing changes in the dynamics. The developed method can suppress instability in 3 seconds, whereas a previous investigation using a steady jet by Deshmukh et al.[28] took more than 10 seconds.

## Conclusions

This study showed successfully implementation of pulsating jets as a control method in closed loop. This type of control method is very effective to control thermo-acoustic instabilities in short duration compared to earlier methods available literatures. To break the coupling between acoustic pressure and unsteady heat release, the designed pulsating mechanism is unable to supply required air in the form of micro-jets in case of pulsating frequency less than 26 Hz. The designed mechanism is not effective in case of pulsating frequency more than 33 Hz as designed mechanism also adding noise in the source at higher frequency. Therefore, there is a need to designed mechanism which can supply required quantity of air without adding any noise in source noise. So, optimisation of reciprocating mechanism dimensions is a point of interest in further studies.

## Ethics statements

Our work did not involve any human subjects, animal experiments or data collection from social media platforms.

## CRediT authorship contribution statement

**Nilaj N. Deshmukh:** Conceptualization, Methodology, Software, Investigation, Writing – review & editing. **Afzal Ansari:** Methodology, Software, Investigation, Writing – review & editing. **Asim P. Tajir:** Methodology, Software, Investigation, Writing – original draft. **Craig C. Almeida:** Methodology, Software, Investigation, Writing – original draft. **Ankita S. Shetty:** . **N.S. Danie:** Methodology, Software, Investigation, Writing – original draft. **Suchita K. Kadam:** Methodology, Software, Investigation, Writing – original draft.

## Declaration of Competing Interest

The authors declare that they have no known competing financial interests or personal relationships that could have appeared to influence the work reported in this paper.

## Data Availability

The data that has been used is confidential.
